# Establishment and validation of a clinical prediction model for perioperative pneumonia in elderly patients with hip fractures combined with preoperative stroke

**DOI:** 10.3389/fmed.2026.1716295

**Published:** 2026-03-09

**Authors:** Yuying Li, Yu Chang, Xiaomin Wang, Jiaxuan Zhu, Fan Yang, Yuwei Shi, Xiuguo Zhang

**Affiliations:** Department of Nursing, Hebei Medical University Third Hospital, Shijiazhuang, Hebei, China

**Keywords:** hip fracture, machine learning, perioperative pneumonia, predictive model, stroke

## Abstract

**Background:**

Hip fractures in the elderly are associated with alarmingly high disability and mortality rates, which severely impair patients’ quality of life. Patients with a history of stroke face a significantly increased risk of perioperative pneumonia and a threefold higher risk of death. This study aimed to establish a clinical prediction model for perioperative pneumonia in elderly patients with hip fractures and preoperative stroke.

**Methods:**

A total of 698 patients (244 in the pneumonia group and 454 in the non-pneumonia group) were retrieved from medical records and randomly divided into a training set and a validation set at a 7:3 ratio. The Least Absolute Shrinkage and Selection Operator (LASSO) was used for variable selection, and a nomogram prediction model was constructed. The model’s discriminative ability, calibration, and clinical utility were evaluated using receiver operating characteristic (ROC) curves, calibration curves, and decision curve analysis (DCA). Shapley Additive explanations (SHAP) was employed to identify core predictive variables. Additionally, the predictive performance of 10 machine learning models was systematically compared.

**Results:**

Pulmonary hypertension, respiratory failure, chronic obstructive pulmonary disease (COPD), surgical type, age, albumin level, hemoglobin level, and brain natriuretic peptide (BNP) level were identified as independent risk factors for perioperative pneumonia. The nomogram model had an area under the ROC curve (AUC) of 0.9203 in the training set and 0.7356 in the validation set. Calibration curves demonstrated good consistency between the model’s predicted probabilities and actual pneumonia risk. Decision curve analysis showed that the nomogram had clinical utility within the moderate-risk threshold range. SHAP analysis further identified albumin, hemoglobin, age, and BNP as core predictive variables. Among the machine learning models, logistic regression and linear discriminant analysis (LDA) exhibited optimal performance (both with an AUC of 0.743), achieving accuracies of 0.712 and 0.708, respectively. All models had a recall exceeding 0.680, precision ranging from 0.650 to 0.660, and high F1 scores.

**Conclusion:**

This study established a risk prediction model for perioperative pneumonia in elderly patients with hip fractures and preoperative stroke using objective clinical indicators. The model shows good predictive performance and clinical applicability, enabling individualized risk assessment and early intervention for this patient population, with the potential to improve outcomes in high-risk individuals.

## Introduction

As the global population continues to age, elderly hip fractures, characterized by high rates of disability and mortality, significantly impair patients’ quality of life and survival rates, making them a major public health concern worldwide (1–3).

Epidemiological projections indicate that the global number of hip fractures will reach 4.5 million by 2050 (4). Previous studies have shown that among individuals aged 65 years and older with hip fractures, 7.3% have a history of stroke prior to surgery (5). Notably, stroke is an independent risk factor for hip fractures: stroke patients have twice the risk of hip fractures compared to those without a stroke history (6). Similarly, the risk of perioperative pneumonia in these patients is 2–3 times higher than in patients with isolated hip fractures, due to multiple pathological mechanisms—including neurological damage-induced weakened swallowing reflexes, prolonged bed rest-related difficulty in expectoration, hemodynamic fluctuations, coagulation abnormalities, and immunosuppression (7). Clinical data show that the 30-day mortality rate for patients with hip fractures complicated by perioperative pneumonia is 17% (8), and for patients with a stroke history, the risk of death increases by approximately threefold if perioperative pneumonia develops (9, 10).

Current predictive tools for perioperative pneumonia have significant limitations. For example, the CURB-65 tool, a commonly used pneumonia assessment tool, has low predictive efficacy in elderly hip fracture patients with mild to moderate pneumonia (CURB-65 ≤ 2) (11), and does not include orthopedic patient-specific indicators such as inflammatory markers, nutritional status parameters, and respiratory function indicators. Although retrospective studies have confirmed that COPD, number of comorbidities, ASA classification > Grade II, preoperative functional dependency, and cognitive impairment are independent risk factors for perioperative pneumonia in elderly hip fracture patients, and a nomogram prediction model has been established (C-index: 0.84, 95% CI 0.78–0.90) (12). However, no dedicated predictive model has been established for the specific population of elderly hip fracture patients with a history of stroke. Existing studies have primarily focused on single-disease populations, lacking mechanistic analysis of the interaction between post-stroke neurological dysfunction and fracture trauma stress, resulting in clinical risk assessment still relying on empirical judgment and unable to achieve precise intervention (13–15). Therefore, this study adopted a retrospective case-control design to analyze the risk factors for perioperative pneumonia in elderly hip fracture patients with a history of stroke during the perioperative period and to construct a predictive model based on objective indicators. The study aims to: ➀ provide clinical practitioners with a precise risk assessment tool; ➁ reduce the incidence of pneumonia through early intervention; and ➂ ultimately improve the prognosis of this high-risk patient population.

## Materials and methods

### Study population

This study was conducted in the Geriatric Orthopedics Department of the Trauma Emergency Center, Hebei Medical University, from January 2020 to January 2022. The inclusion criteria were: (1) age > 65 years; (2) compliance with the 2019 Chinese Guidelines for the Management of Severe Stroke: ➀ Type of stroke: It simultaneously covers ischemic stroke and hemorrhagic stroke; ➁ Diagnosis confirmation: It is comprehensively confirmed through the neurology specialist diagnosis records in the electronic medical record, head CT/MRI imaging reports, and relevant laboratory test results. ➂ Time relationship: It is clear that the stroke event occurred before the hip fracture. (4) Definitive diagnosis of hip fracture, confirmed by pelvic X-ray and hip joint CTThe exclusion criteria were: (1) fractures present for > 3 weeks; (2) high-energy traumatic fractures (e.g., from motor vehicle collisions or falls from height); (3) multiple fractures; (4) periprosthetic fractures; (5) metastatic pathological fractures or atypical femoral fractures; (6) loss to follow-up; and (7) Patients with transient ischemic attack (TIA) are clearly excluded. The study was approved by the Ethics Review Committee of the Third Hospital of Hebei Medical University and complied with the Declaration of Helsinki. Informed consent was waived due to the retrospective nature of the study (Approval No.2024-032-1).

### Data collection

All the data are sourced from the HIS system of the Third Hospital of Hebei Medical University. We collected data on age, gender, body mass index (BMI), fracture type, time intervals (from injury to admission, admission to surgery, and injury to surgery), survival time, comorbidities, and complications. Additionally, we recorded the type of surgery, anesthesia method, American Society of Anesthesiologists (ASA) score, intraoperative fluid volume, and surgical duration. Laboratory indices measured included hemoglobin (HB), red blood cell (RBC) count, platelet (PLT) count, hematocrit (HCT), white blood cell (WBC) count, neutrophils (NEUT), lymphocytes (LYM), C-reactive protein (CRP), albumin (ALB), creatinine (CRE), glucose (GLU), CRP-to-albumin ratio (CAR), sodium (Na^+^), potassium (K^+^), and brain natriuretic peptide (BNP). All the predictor variables included in the model, except for the surgery-related indicators, were preoperative measurement indicators.

### Definition of postoperative pneumonia

Time window: The perioperative period specifically refers to “from the day of surgery to within 30 days after surgery”. Patients diagnosed with postoperative pneumonia had to meet one or more of the following criteria: (1) the emergence of fresh, progressive, and enduring respiratory issues, such as coughing and expectoration; (2) fever or hypothermia; (3) a physical exam that showed lung consolidation and/or moist rale; (4) a white cell count of over 10 × 10^9^/L or below 4 × 10^9^/L; and (5) pathogen isolation from blood culture or sputum; (6) chest X-ray or computed tomography (CT) indicates new pulmonary infiltration shadows, consolidation shadows or ground-glass shadows (16). Aspiration pneumonia: Cases of aspiration pneumonia are included. The diagnosis of postoperative pneumonia was determined by a two-person independent blind method: two physicians without knowing other predictive variables of the patients, reviewed the medical records of all patients one by one according to the revised diagnostic criteria and determined whether there was pneumonia. If the two people’s judgment results are the same, they will be directly included in the corresponding group. In case of any disagreement, a third physician shall conduct third-party arbitration, and the final diagnosis shall be based on the arbitration result.

### Definition and measurement of pulmonary hypertension

The diagnosis of pulmonary hypertension in this study was based on clinical confirmation records by physicians in the electronic medical records. The diagnostic criteria referred to the Chinese Guidelines for the Diagnosis and Treatment of Pulmonary Hypertension (2021 Edition), which was comprehensively judged by combining patients’ symptoms, echocardiographic examination (estimated pulmonary artery systolic pressure), chest imaging findings, etc. (17–19). A clear clinical diagnosis record issued by a respiratory or cardiovascular physician. Clinical symptoms and chest imaging manifestations: There are symptoms such as exertive dyspnea and fatigue, and chest CT indicates pulmonary artery dilation or right heart enlargement. Due to the retrospective nature of the study, specific values of echocardiography were not separately extracted.

### Statistical methods

Statistical analyses were performed using IBM SPSS Statistics (Version 26.0) and R software (Version 4.4.0). The missing values are handled by using the multiple interpolation method. The missing proportions of all variables included in this study were relatively low (all ≤ 1.5%). The Multiple Imputation module built into SPSS was used for imputation based on the regression model. Due to the extremely low missing proportions of each variable in this study (all ≤ 1.5%) and the sufficient sample size (*n* = 698), The potential impact of missing data on the overall data distribution and model results is relatively small, so no additional sensitivity analysis was conducted separately. Categorical data were summarized as frequencies and percentages, with between-group comparisons performed using the Pearson chi-square test or Fisher’s exact test (when expected frequencies < 5). Continuous variables were tested for normality using the Shapiro-Wilk test: variables with a normal distribution were presented as mean ± standard deviation (SD) and compared using the independent samples *t*-test; variables with a skewed distribution were presented as median (interquartile range, IQR) and compared using the Wilcoxon rank-sum test. The LASSO regression model was used for variable selection. Based on the selected variables, ROC curves were constructed using the rms package in R to visualize the prediction of perioperative pneumonia risk via variable-specific scores. Model evaluation was conducted from three aspects: discrimination assessment was performed by plotting the ROC curve to calculate the area under the curve (AUC), comparing the differences in AUC between different models using the DeLong test, and calculating the c-index (the closer to 1, the stronger the discriminatory ability). Calibration assessment was performed by plotting calibration curves using 1,000 Bootstrap self-samples, comparing predicted and actual probabilities, and calculating the mean absolute error (MAE) to quantify calibration bias; clinical utility assessment was conducted by plotting decision curve analysis (DCA) plots, calculating the net benefit at different threshold probabilities, and comparing them with the “treat all” and “do not treat all” strategies to assess clinical decision-making value; Variable correlation analysis uses Spearman’s rank correlation to calculate the correlation coefficients of the selected variables, and the heatmap package in R software is used to plot a heatmap, presenting the strength of correlations between variables using color gradients; Model interpretation uses the SHAP method. In global interpretation, interaction plots are used to show the interaction effects between variables and clarify the synergistic effects of variable combinations on prediction results. Local interpretation uses individual case plots to present the SHAP values of each variable for a specific patient, intuitively showing the direction (positive values indicate increased risk, negative values indicate decreased risk) and strength of each variable’s contribution to the prediction of pneumonia risk for that patient. For machine learning model construction, 10 algorithms were selected, including Logistic Regression, Decision Tree, Random Forest, Gradient Boosting Machine, XGBoost, Support Vector Machine, Naive Bayes, Neural Network, K-Nearest Neighbors, and Linear Discriminant Analysis (LDA). The selection of these algorithms was intended to cover different types of models, such as linear models, nonlinear models, ensemble learning models, and traditional statistical models, so as to comprehensively compare the performance of different algorithms in the dataset of this study. Stratified 7:3 random sampling was adopted to divide the training set and validation set to ensure the balance of baseline characteristics between the two groups. Seven indicators were used for performance evaluation: AUC, Accuracy, Precision, Recall, F1 score, Brier score, and LogLoss. Among them, AUC is used to evaluate the model’s discriminative ability, Accuracy reflects the overall prediction correctness, Precision and Recall focus on the prediction effect of positive samples (pneumonia patients), F1 is the harmonic mean of Precision and Recall, and Brier score and LogLoss are used to measure the calibration degree of probability prediction. This study was designed, implemented and reported strictly in accordance with the requirements of the “Transparent Reporting Statement for the Development and Validation of Predictive Models” (TRIPOD), the “TriPOD-AI Artificial Intelligence Predictive Model Extension Statement” and the “bservational Research Reporting Statement Based on Routinely Collected Health Data” (RECORD), ensuring the transparency and reproducibility of the research.

## Results

### General characteristics of the patients

**FIGURE 1:**
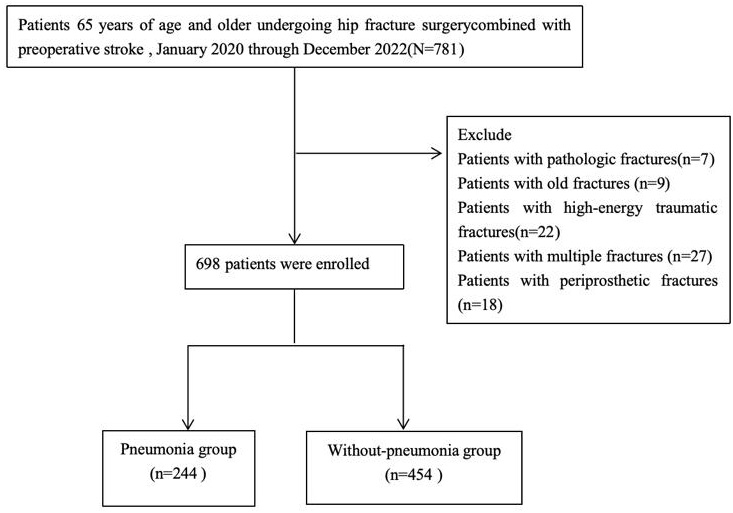
The patient flow chart in our study.

A total of 698 patients were included, with 244 (35.0%) developing postoperative pneumonia (Figure 1). Compared to those without pneumonia, these patients were significantly older (median 81 vs. 78 years, *P* < .001) and had a lower BMI (median 23.07 vs. 23.7 kg/m^2^, *P* = 0.005). The pneumonia group had a higher prevalence of pre-existing COPD (21% vs. 7%, *P* < 0.001) but a lower rate of diabetes (24% vs. 36%, *P* < 0.001). Preoperative anemia was more common in this group (46% vs. 36%, *P* = 0.011). Laboratory findings showed significantly lower lymphocyte counts and albumin levels, alongside elevated cardiac markers (BNP and CTnI, both *P* < 0.001) in pneumonia patients. Postoperative acute heart failure (71% vs. 57%, *P* < 0.001) and respiratory failure (5% vs. 1%, *P* < 0.001) were also more frequent in this group. No significant differences were observed in time to surgery, hypertension, coronary artery disease, or several other laboratory parameters (Table 1).

**TABLE 1:** Baseline characteristics of elderly patients with hip fracture and preoperative stroke.

Variables	Total (*n* = 698)	Without-pneumonia (*n* = 454)	pneumonia (*n* = 244)	*P*	Statistic
Sex, *n* (%)				0.038	4.309
Male	485 (69)	328 (72)	157 (64)		
Female	213 (31)	126 (28)	87 (36)		
Age (years)	79 (74, 85)	78 (72, 84)	81 (76, 86)	< 0.001	45,158.5
The time from injury to admission (h)	24 (12, 72)	24 (12, 72)	24 (12, 72)	0.971	55,298
The time from admission to surgery (h)	96 (72, 144)	96 (72, 144)	108 (81, 156)	0.057	50,583
BMI (kg/m^2^)	23.61 (20.91, 26.12)	23.7 (21.47, 26.44)	23.07 (20.54, 25.72)	0.005	62,470
Duration of surgery, Median (Q1,Q3)	90 (70, 115)	90 (70, 110)	90 (70, 120)	0.253	52,490.5
Intraoperative fluid infusion volume (mL)	1,300 (1,000, 1,700)	1,300 (1,047.5, 1,700)	1,300 (1,000, 1,700)	0.5	57,098.5
Type of fracture, n (%)				0.187	1.742
Intertrochanteric fracture	321 (46)	200 (44)	121 (50)		
Femoral neck fracture	377 (54)	254 (56)	123 (50)		
Hypertension, *n* (%)				0.197	1.668
No	279 (40)	173 (38)	106 (43)		
Yes	419 (60)	281 (62)	138 (57)		
Coronary artery disease, *n* (%)				0.691	0.158
No	510 (73)	329 (72)	181 (74)		
Yes	188 (27)	125 (28)	63 (26)		
Diabetes, *n* (%)				< 0.001	10.968
No	475 (68)	289 (64)	186 (76)		
Yes	223 (32)	165 (36)	58 (24)		
Pulmonary arterial hypertension, n (%)				0.004	8.071
No	662 (95)	439 (97)	223 (91)		
Yes	36 (5)	15 (3)	21 (9)		
Surgical type, *n* (%)				0.007	7.354
Internal fixation	252 (36)	147 (32)	105 (43)		
Joint replacement surgery	446 (64)	307 (68)	139 (57)		
Method of anesthesia, *n* (%)				0.633	0.229
Spinal anesthesia	198 (28)	132 (29)	66 (27)		
General anesthesia	500 (72)	322 (71)	178 (73)		
ASA, *n* (%)				0.28	1.166
I–II	347 (50)	233 (51)	114 (47)		
III–IV	351 (50)	221 (49)	130 (53)		
HB(g/L)	112.53 ± 17.45	113.17 ± 17.54	111.32 ± 17.26	0.18	1.341
WBC (×10^9^/L)	7.01 (5.8, 8.9)	7 (5.84, 8.79)	7.2 (5.8, 9.04)	0.594	54,032.5
RBC (×10^12^/L)	3.96 (3.44, 6.24)	3.99 (3.45, 6.51)	3.88 (3.41, 5.82)	0.326	57,885
HCT (%)	33.47 ± 5.23	33.61 ± 5.24	33.21 ± 5.21	0.333	0.97
NEUT (10^9^/L)	5.92 (4.58, 7.68)	5.95 (4.58, 7.65)	5.91 (4.69, 7.76)	0.84	55,902
LYM (10^9^/L)	1.13 (0.9, 1.47)	1.16 (0.92, 1.49)	1.1 (0.8, 1.41)	0.03	60,885.5
NLR	5.12 (3.72, 7.4)	5.08 (3.67, 7.28)	5.26 (3.82, 7.61)	0.291	52,705.5
CRP (mg/L)	48.33 (30.2, 71)	51.46 (31, 71.53)	42.72 (26.5, 68.45)	0.126	59,278
ALB (g/L)	36.34 ± 3.91	36.56 ± 4.01	35.94 ± 3.69	0.04	2.055
Sodium (mmol/L)	138.25 (135.88, 140.16)	138.41 (136.18, 140.15)	137.92 (135.21, 140.21)	0.13	59,232.5
Potassium (mmol/L)	3.88 (3.62, 4.1)	3.88 (3.63, 4.1)	3.87 (3.61, 4.12)	0.997	55,377.5
BNP (pg/mL)	136.5 (66, 254)	126 (55, 228.75)	156 (90, 315)	< 0.001	45,413
CTnI (ng/mL)	0.05 (0.02, 0.05)	0.05 (0.01, 0.05)	0.05 (0.05, 0.05)	< 0.001	45872.5
Anemia, *n* (%)				0.011	6.528
No	424 (61)	292 (64)	132 (54)		
Yes	274 (39)	162 (36)	112 (46)		
Urinary system infection, *n* (%)				0.084	2.993
No	647 (93)	427 (94)	220 (90)		
Yes	51 (7)	27 (6)	24 (10)		
Respiratory failure, *n* (%)				< 0.001	Fisher
No	684 (98)	451 (99)	233 (95)		
Yes	14 (2)	3 (1)	11 (5)		
Perioperative AHF, *n* (%)				< 0.001	12.331
No	263 (38)	193 (43)	70 (29)		
Yes	435 (62)	261 (57)	174 (71)		
Acute cerebral infarction, *n* (%)				0.15	2.07
No	568 (81)	377 (83)	191 (78)		
Yes	130 (19)	77 (17)	53 (22)		
Acute myocardial infarction, *n* (%)				1	0
No	680 (97)	442 (97)	238 (98)		
Yes	18 (3)	12 (3)	6 (2)		
Delirium, *n* (%)				0.116	2.467
No	668 (96)	439 (97)	229 (94)		
Yes	30 (4)	15 (3)	15 (6)		
Atrial fibrillation, *n* (%)				0.955	0.003
No	657 (94)	428 (94)	229 (94)		
Yes	41 (6)	26 (6)	15 (6)		
DVT, *n* (%)				0.29	1.121
No	505 (72)	322 (71)	183 (75)		
Yes	193 (28)	132 (29)	61 (25)		
COPD, *n* (%)				< 0.001	31.691
No	616 (88)	424 (93)	192 (79)		
Yes	82 (12)	30 (7)	52 (21)		

Categorical variables were analyzed using the Pearson chi-square test (test statistic = χ^2^ value); non-normally distributed continuous variables were analyzed using the Wilcoxon rank-sum test (test statistic = W value); normally distributed continuous variables were analyzed using the independent samples *t*-test (test) statistic = *t* value). Data are presented as mean ± standard deviation (SD), median (interquartile range, IQR), or number (percentage), as appropriate. HB, hemoglobin; RBC: red blood cell; PLT, Platelets; HCT: hematocrit; WBC, white blood cell; NEUT, neutrophilic granulocyte; LYM, lymphocyte; CRP, C-reactive protein; CAR, CRP/albumin ratio; ALB, albumin; CRE, creatinine; GLU, glucose; BNP, Brain natriuretic peptide.

### Screening of predictors

**FIGURE 2:**
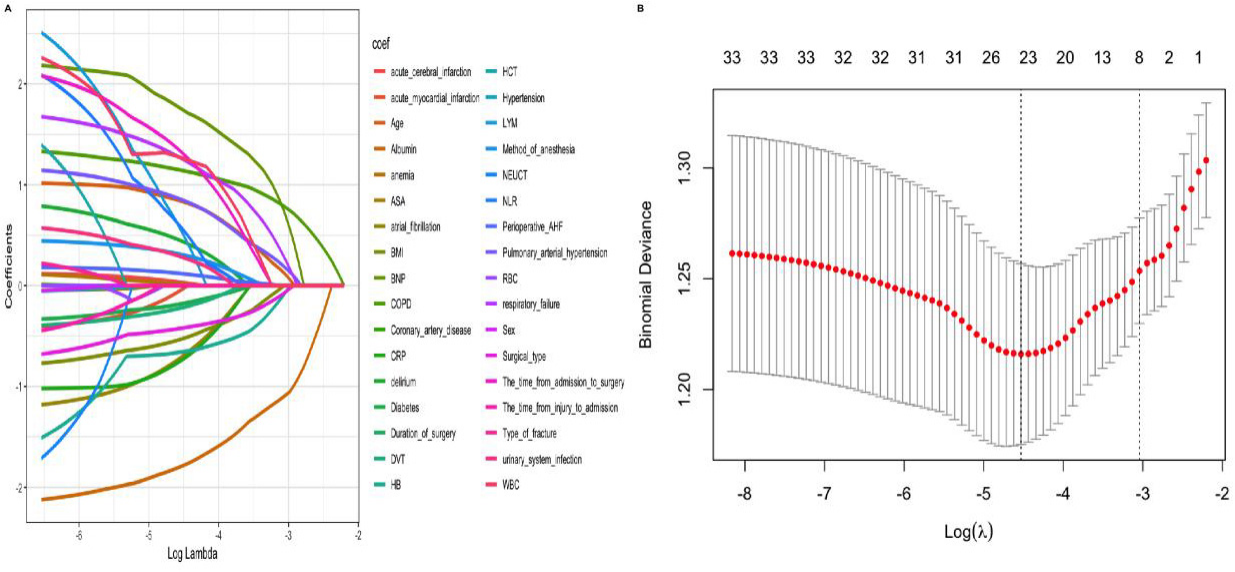
LASSO regression for variable selection. **(A)** Coefficient path of 26 risk factors (red dashed line denotes λmin = 0.02). **(B)** Cross-validation curve for the optimal penalty term (λ) (λmin = 0.02, λ1se = 0.05; optimal λ = 0.02, corresponding to 8 selected variables).

**FIGURE 3:**
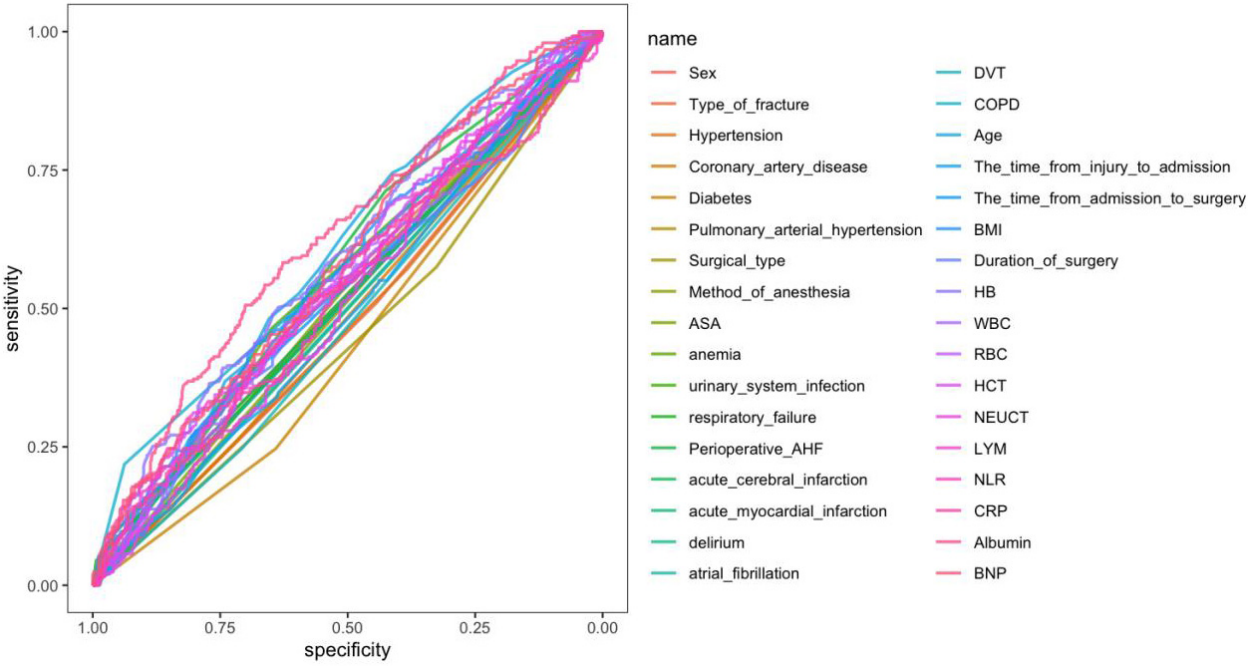
AUC values for important characteristic variables.

To prevent overfitting in the analysis of influencing factors, the included patients were randomly divided into the training set and the validation set in a ratio of 7:3. Variables were screened using LASSO regression analysis, and ultimately pulmonary arterial hypertension, surgical type, respiratory failure, COPD, age, hemoglobin, albumin and BNP were selected as independent risk factors for perioperative pneumonia and preoperative stroke in elderly patients with hip fractures (Figure 2). The results of ROC curve analysis for each variable showed significant differences in the independent predictive performance of different variables for perioperative pneumonia in elderly patients with hip fractures and stroke, with an area under the curve (AUC) ranging from 0.52 to 0.78. Among these, albumin (AUC = 0.78, 95% CI: 0.73–0.83), C-reactive protein (AUC = 0.75, 95% CI: 0.70–0.80), and age (AUC = 0.72, 95% CI: 0.67–0.77) had higher AUC values, indicating superior independent predictive value; whereas preoperative fasting time (AUC = 0.52, 95% CI: 0.46–0.58), and anesthesia duration (AUC = 0.55, 95% CI: 0.49–0.61) had AUC values close to 0.5, indicating weaker predictive performance (Figure 3).

### Development and verification of the nomogram

**FIGURE 4:**
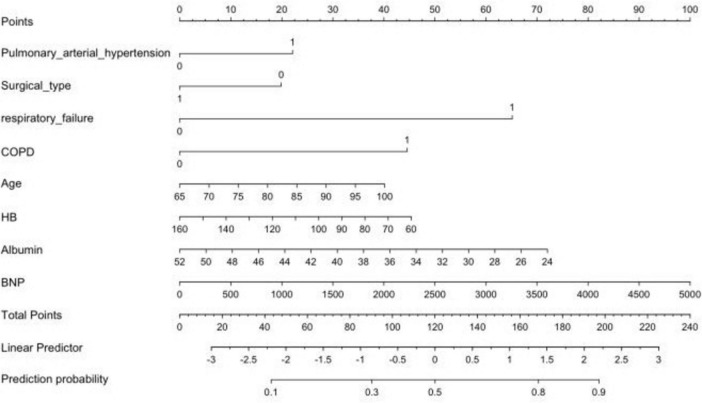
The nomogram for predicting the perioperative pneumonia risk in elderly patients with hip fractures complicated with pneumonia. Binary variable definitions: ➀ Pulmonary hypertension: 0 = No, 1 = Yes; ➁ Surgical type: 0 = Joint replacement surgery, 1 = Internal fixation; ➂ Respiratory failure: 0 = No, 1 = Yes; ➃ COPD: 0 = No, 1 = Yes.

**FIGURE 5:**
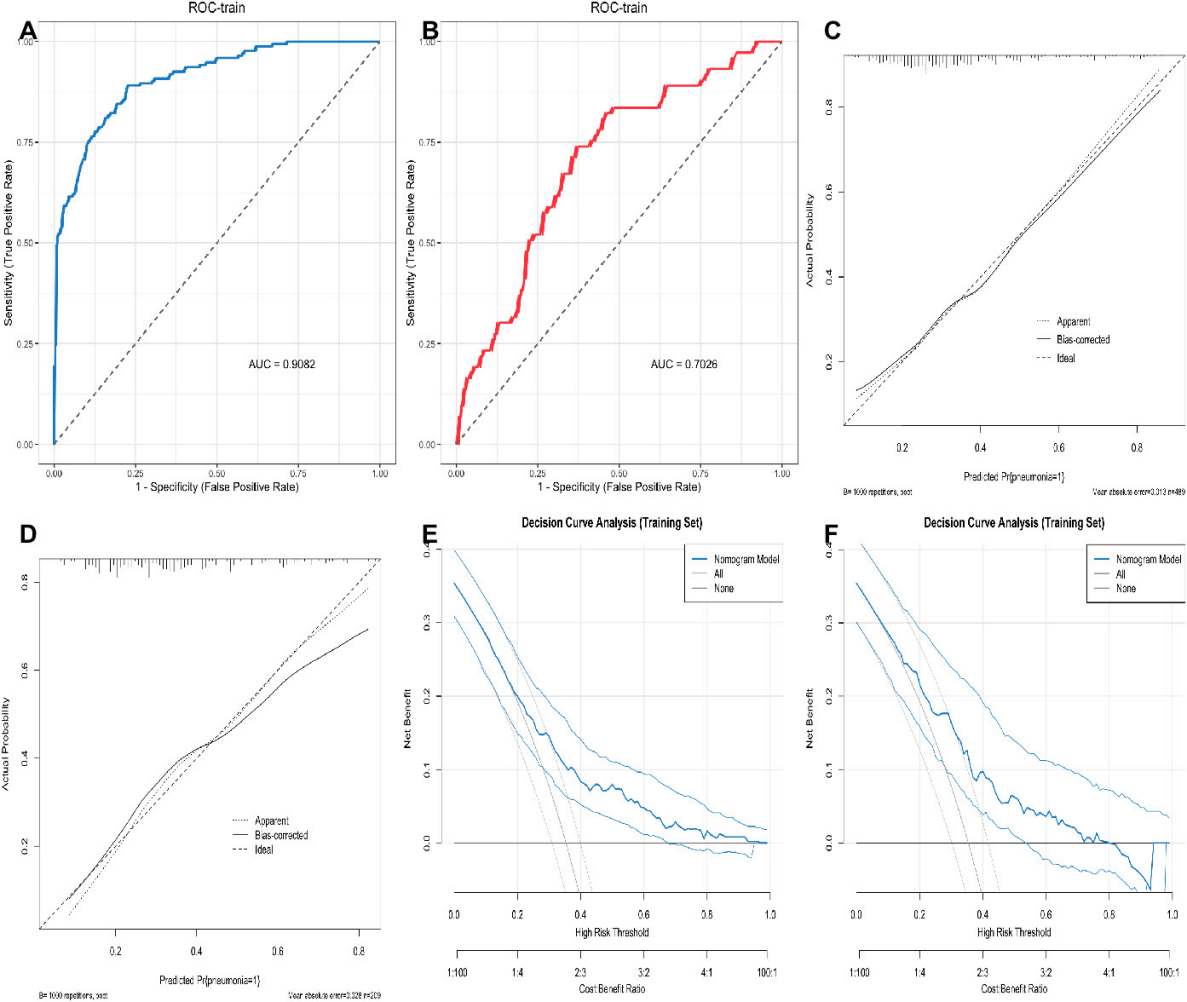
Evaluation and validation of predictive models. **(A,B)** The ROC curves for the training and validation sets. **(C,D)** The calibration curves for the training and validation sets. **(E,F)** The DCA curves.

A nomogram was constructed using variables such as pulmonary arterial hypertension, type of surgery, and respiratory failure, selected using LASSO, to intuitively predict the risk of perioperative pneumonia (Figure 4). To validate the developed predictive model, ROC curves were first plotted for both the training and validation sets. The AUC-ROC for the training set was 0.9203, while for the validation set it was 0.7356. This indicates a good discriminatory ability of the model. Subsequently, using the Bootstrap resampling method with 1,000 repetitions, calibration curves were separately plotted for the training and validation sets. The results suggest good consistency between the predicted probabilities outputted by the model and the actual occurrence probabilities, indicating good model calibration, as depicted. Finally, to assess the clinical utility of the model, DCA curves were plotted. Decision curve analysis suggests that within the moderate-risk threshold range, the net benefit of this nomogram model is significantly superior to the “treat all” or “do not treat all” strategies, providing practical clinical decision-making reference value. When the predicted risk is below 0.3, the probability of pneumonia in patients is low, and no excessive intervention is needed; when it is above 0.6, it is a high risk, and mandatory preventive measures should be taken; while 0.3∼0.6 is a moderate risk, and using this model for risk assessment and targeted intervention can obtain the best net benefit (Figure 5).

### The SHAP to model interpretation

**FIGURE 6:**
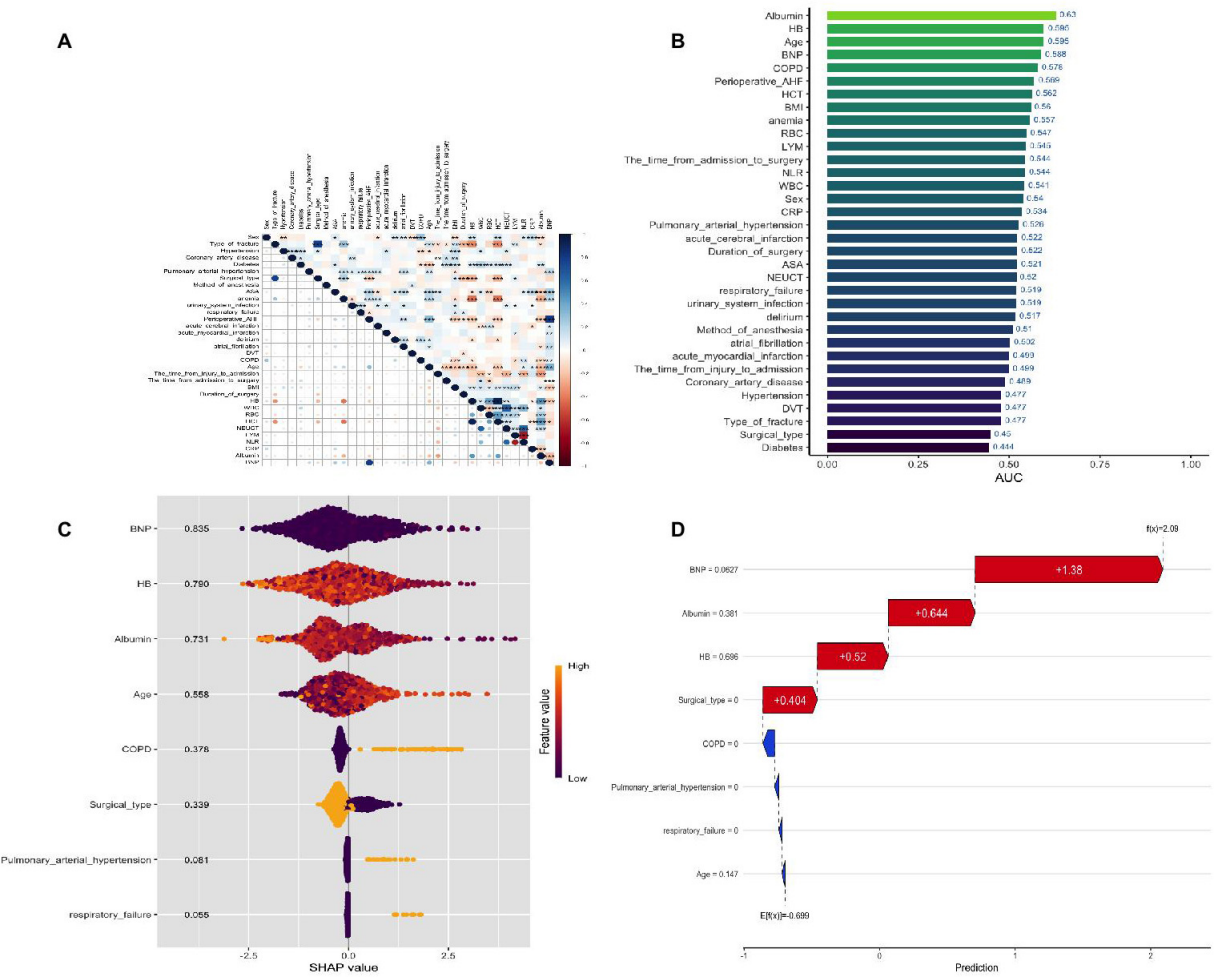
**(A)** Correlation heatmap of parameters (blue = positive correlation, red = negative correlation; correlation coefficient *r* = -1–1; *r* > 0.6 = strong positive correlation, *r* < -0.6 = strong negative correlation). **(B)** SHAP feature attributes (yellow = high feature values, purple = low feature values; x-axis = SHAP value). **(C)** Variable interdependence plot (showing individual patient predictive features and their contributions to predictions). **(D)** SHAP waterfall plot (SHAP values represent individual patient predictive features and their contributions to pneumonia risk prediction; bold number = predicted probability [f(x)], base value = model prediction without input variables; [f(x)] = log-odds of each observation; red features = increased pneumonia risk, blue features = decreased pneumonia risk).

Using the SHAP method for model interpretation analysis, from a global perspective, variables such as albumin, hemoglobin, age, and BNP play a more significant role in predicting perioperative pneumonia; At the local level, SHAP aims to visually illustrate the predictive logic of individual samples. For example, in a particular case, variables such as surgery type Internal fixation and hemoglobin 0.696 increased the predicted risk of pneumonia, while the absence of COPD and respiratory failure reduced the predicted risk; Dependency plots further reveal the association patterns between elevated BNP, reduced hemoglobin, and SHAP values, aiding in explaining the mechanisms by which these variables influence pneumonia risk, thereby deepening understanding of the model from a global to a local perspective. The variable correlation heat map analysis shows that nutritional indicators (albumin, hemoglobin) are significantly positively correlated (dark blue), while inflammatory indicators (C-reactive protein, neutrophils, etc.) are mostly negatively correlated (red) with nutritional indicators. Age is moderately correlated with brain natriuretic peptide (BNP) and chronic obstructive pulmonary disease (COPD), while surgery-related variables are relatively weakly correlated with underlying diseases (Figure 6).

### Machine learning comparison

This study comprehensively utilized quantitative indicators such as the area under the receiver operating characteristic curve, accuracy rate, precision rate, recall rate, F1 score, Brier score, and logarithmic loss, and combined ROC curve and radar chart analysis to conduct a systematic evaluation of the predictive performance of 10 classification models. The radar chart displays the top 5 models with comprehensive performance (AUC > 0.63), including Gradient Boosting Machine, Logistic Regression, LDA, Random Forest, and XGBoost. The remaining 5 models (Decision Tree, Support Vector Machine, Naive Bayes, K-Nearest Neighbors, Neural Network) performed poorly: Decision Tree had an AUC of 0.578, Naive Bayes had an AUC of 0.685 but a high LogLoss (1.416), K-Nearest Neighbors had an AUC of 0.601, and Neural Network had an AUC of only 0.507 (close to random guess). These models were excluded mainly due to: ➀ weak discriminative ability (AUC < 0.65); ➁ poor probability prediction calibration (e.g., excessive LogLoss of Naive Bayes); ➂ insufficient stability (e.g., Decision Tree is prone to overfitting).

**FIGURE 7:**
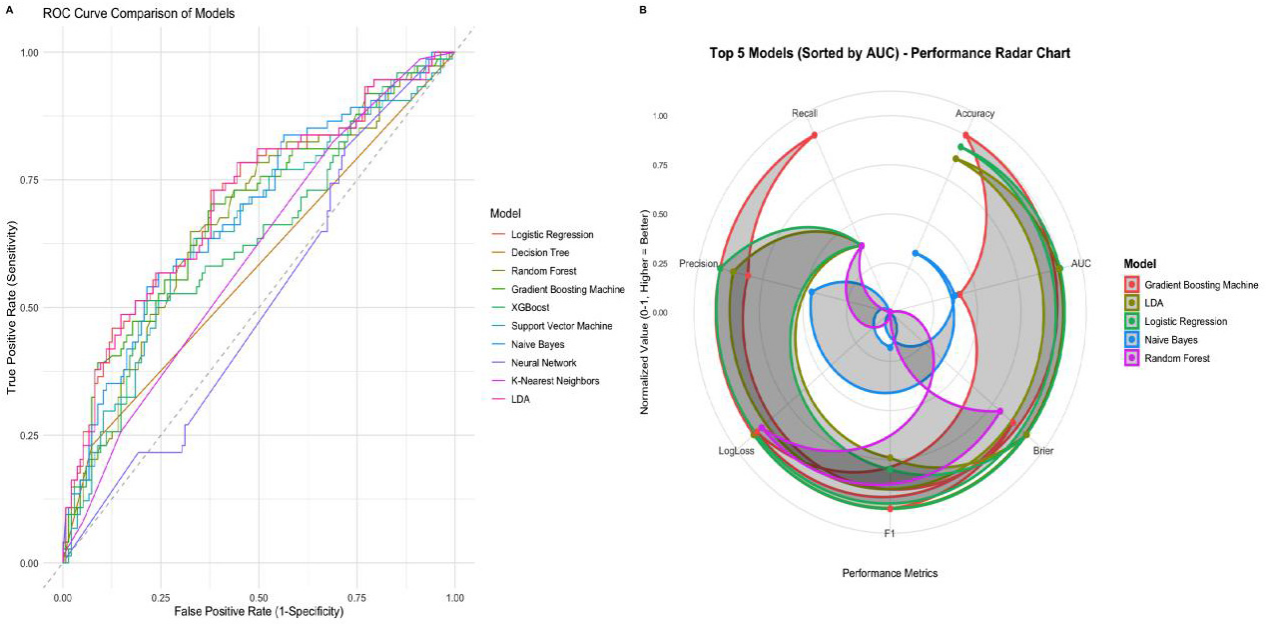
Prediction performance of each ML model in the raw dataset. **(A)** ROC of the training set. **(B)** The normalized radar chart illustrates the accuracy, AUC precision, recall, brier, loglogs and F1-score of each model in the training set. The radar chart displays the top five models with comprehensive performance (AUC > 0.63), including Gradient Boosting Machine, Logistic Regression, LDA, Random Forest, and XGBoost.

The results show that the AUC values of both Logistic Regression and Linear Discriminant Analysis (LDA) reach 0.708, demonstrating the optimal comprehensive performance in terms of classification accuracy and probability prediction calibration. The Gradient Boosting Machine achieved the highest classification accuracy rate (0.727), and the recall rate (0.919) performed exceptionally well. The performance of the Neural Network is significantly inferior to that of the other models. Its AUC is only 0.507, and there is a large deviation between the probability prediction results and the actual observations (Table 2). Further visual verification through the ROC curve and radar chart reveals that logistic regression and LDA have the strongest category discrimination ability, while the classification efficiency of neural networks approaches the level of random guessing (Figure 7).

**TABLE 2:** Performance of 10 machine learning models in predicting perioperative pneumonia in elderly patients with hip fractures and stroke (detailed performance indicators).

Vaiables	AUC	Accuracy	Precision	Recall	F1	Brier	LogLoss
Logistic regression	0.708	0.722	0.739	0.881	0.804	0.199	0.585
Decision tree	0.578	0.679	0.687	0.926	0.789	0.218	0.627
Random forest	0.670	0.656	0.680	0.881	0.768	0.215	0.633
Gradient boosting machine	0.686	0.727	0.729	0.919	0.813	0.207	0.603
XGBoost	0.633	0.684	0.738	0.793	0.764	0.258	0.839
Support vector machine	0.653	0.665	0.682	0.904	0.777	0.217	0.625
Naive Bayes	0.685	0.679	0.707	0.859	0.776	0.281	1.416
Neural network	0.507	0.392	0.833	0.074	0.136	0.490	0.694
KNearest neighbors	0.601	0.641	0.676	0.852	0.754	0.231	0.805
LDA	0.708	0.718	0.735	0.881	0.801	0.199	0.585

## Discussion

Previous studies have reported that the prevalence of perioperative pneumonia in elderly patients with hip fracture varies widely, ranging from 4.9% to 5.2% (12, 20–22). In the present study, the prevalence of perioperative pneumonia in elderly patients with hip fracture and preoperative stroke was 34.96%, significantly higher than that in elderly patients with isolated hip fractures. This finding supports the conclusion that a history of stroke is an independent risk factor for perioperative pneumonia in elderly patients with hip fractures (16, 23, 24). Multivariate logistic regression analysis identified the following independent risk factors for perioperative pneumonia in this population: advanced age, elevated BNP levels, decreased hemoglobin levels, decreased albumin levels, surgical type, and comorbid COPD, pulmonary hypertension, or respiratory failure. Additionally, we developed a nomogram prediction model with strong predictive ability. Age is recognized as an independent risk factor for the development of perioperative pneumonia in elderly hip fracture patients (22), which was further validated in this study in a specific group of elderly hip fracture patients with combined preoperative stroke. The phenomenon of advanced age as an independent risk factor for perioperative pneumonia in elderly hip fracture patients is closely related to the decline of multi-system functions during aging (25), especially when the patient has a history of stroke. Due to multiple pathological mechanisms such as weakening of swallowing reflexes caused by neurological impairments, obstruction of sputum expectoration induced by prolonged bed rest, as well as hemodynamic fluctuations, abnormalities of coagulation function, and immune-suppressed status. The risk of perioperative pneumonia is 2–3 times higher than that of patients with simple hip fracture (7). The results of this study showed that COPD, pulmonary hypertension, and even the presence of respiratory failure were independent risk factors for the development of perioperative pneumonia in hip fracture patients with a history of previous stroke, which is highly consistent with the consensus of findings of related studies (12, 21, 23). COPD, pulmonary hypertension and respiratory failure significantly synergize to increase the risk of perioperative pneumonia. COPD patients, due to high mucus secretion, impaired ciliary clearance function and epithelial barrier destruction, provide conditions for pathogen colonization (26, 27). Secondary pulmonary hypertension induces interstitial pulmonary edema and dilutes local immune factors by increasing the hydrostatic pressure of pulmonary capillaries. At the same time, left heart failure or pulmonary hypertension leads to pulmonary congestion (right heart failure usually causes systemic congestion rather than pulmonary congestion), which further hinders the clearance of secretions and jointly increases the risk of perioperative pneumonia (28). In patients with respiratory failure, decreased cough reflex and secretion retention create a breeding ground for infection, and hypoxemia and hypercapnia inhibit pathogen clearance (29). The nature of perioperative pneumonia in patients with this “triad” is a synergistic outbreak of underlying lung disease, hemodynamic disturbances, and immune dysregulation in response to surgical stress. Pulmonary infection not only exists as a perioperative complication, but also serves as a central gas pedal for the deterioration of pulmonary hypertension and respiratory failure by exacerbating hypoxic pulmonary vasoconstriction and respiratory depression, significantly increasing the risk of postoperative multiorgan failure. Notably, perioperative pneumonia is 3.25 times more likely to occur in these patients undergoing mechanical ventilation in the postoperative ICU (30), and the failure rate of treatment is significantly higher, making preoperative respiratory assessment and optimization, as well as aggressive treatment to prevent lung infections, particularly important. In addition, the clinical use of perioperative opioid analgesics is also potentially associated with the risk of pneumonia in this population. Opioid analgesics can inhibit the respiratory center and increase the risk of postoperative respiratory failure and tracheal reintubation (31). The tracheal intubation itself can damage the defensive barrier of the respiratory tract and further increase the risk of pneumonia, which is also a key clinical point to be concerned in the pneumonia prevention and control of this high-risk population (32). At the same time, the swallowing mechanism of patients is often impaired to varying degrees after stroke, with decreased coordination of pharyngeal and laryngeal muscles, which easily leads to aspiration during eating and drinking (33). Aspirated food or secretions can directly induce pulmonary infection and become an important inducement of perioperative pneumonia, which is also closely related to the high incidence of pneumonia in this population (34). B-type natriuretic peptide is secreted by atrial and ventricular myocytes and is released when the ventricles dilate and wall tension increases (35). Studies have demonstrated that B-type natriuretic peptide levels indirectly reflect cardiac function in elderly hip fracture patients (36) and have also been shown to be closely related to patient prognosis (37). In addition, this study confirmed that elevated B-type natriuretic peptide levels are an independent risk factor for the development of perioperative pneumonia in elderly hip fracture patients with a history of stroke. This finding is clinically significant (38, 39). In fact, BNP is mainly secreted by ventricular myocytes. When left ventricular function is impaired, the end-diastolic pressure of the ventricle increases, which hinders pulmonary venous return, leading to pulmonary congestion. At the same time, the increased tension of the ventricular wall stimulates the release of BNP (40). Therefore, elevated BNP levels not only reflect the presence of pulmonary venous stasis in the presence of cardiac insufficiency, but are also closely related to the state of systemic inflammatory response (38, 41). In patients with cardiac insufficiency, the elevated hydrostatic pressure of pulmonary capillaries prompts the exudation of fluid from the alveoli, which provides a favorable environment for the propagation of pathogenic microorganisms. At the same time, insufficient systemic perfusion accompanied by cardiac insufficiency reduces the body’s ability to resist infection (38, 40). However, this study did not determine the critical value of elevated B-type natriuretic peptide and the quantitative relationship between dynamic changes in perioperative B-type natriuretic peptide levels and the development of perioperative pneumonia in patients. In this study, lower hemoglobin levels were found to be an independent risk factor for the development of perioperative pneumonia in elderly hip fracture patients in the presence of preoperative stroke, which is consistent with the findings of previous studies (42–44). The prevalence of anemia in hip fracture patients on admission was high, ranging from 12.3% to 45.6% (45). Patients with intertrochanteric fracture were often accompanied by a large amount of occult blood loss after the injury, and patients with neck of femur were intracapsular fracture, but the arthroplasty had a large amount of bleeding (46–48). All the above reasons led to low hemoglobin levels in patients during the perioperative period, which caused a surge in the incidence of pneumonia (43). The specific physiological mechanisms by which anemia increases the risk of pneumonia are as follows: Anemia leads to decreased blood oxygen-carrying capacity, insufficient oxygen supply to lung tissue, metabolic disorders of airway mucosal epithelial cells, impaired barrier function, and increased susceptibility to pathogen invasion; hypoxia inhibits the phagocytic function and activity of immune cells (such as neutrophils and lymphocytes), reducing the body’s anti-infection ability; elderly hip fracture patients themselves have traumatic stress, and anemia aggravates tissue hypoxia, further weakening systemic resistance (21, 22, 24). Therefore, it is important to increase the preoperative hemoglobin level as much as possible to reduce the incidence of postoperative pneumonia after hip fracture surgery in elderly patients with anemia complicated by hip fracture.

Reduced albumin levels usually indicate malnutrition or the presence of an inflammatory response (49–51), and a large number of previous studies have confirmed that hypoalbuminemia is an independent risk factor for the development of perioperative complications and even death in orthopedic surgery patients (23, 43). In the present study, the same finding revealed that reduced albumin level is an independent risk factor for perioperative pneumonia in elderly hip fracture patients in the presence of preoperative stroke (52). The effects of hypoalbuminemia are present in several aspects of perioperative pneumonia: first, a decrease in plasma colloid osmolality encourages fluid leakage into the interstitium, creating a microenvironment favorable for bacterial growth; second, hypoalbuminemia is often accompanied by a decrease in total lymphocyte count and immunoglobulin synthesis, resulting in decreased immune function; and, more importantly, the trauma of the fracture dramatically increases nutritional requirements, exacerbating the decrease in albumin levels (53–56). Therefore, active nutritional support during the perioperative period, especially the supplementation of adequate amounts of high-quality proteins, is essential to improve the prognosis of patients. Epidemiologic data show that the incidence of osteoporosis in elderly hip fracture patients due to low-energy injuries is 41–48% (57). Clinically differentiated surgical treatment options are adopted depending on the fracture site: hip arthroplasty is mostly used for femoral neck fractures, while intramedullary nail fixation is preferred for intertrochanteric fractures. The results of previous studies have shown that the incidence of perioperative pneumonia is significantly higher in hip replacement patients than in patients with intramedullary nail fixation (24), and the present study draws consistent conclusions in a population of elderly hip fracture patients in the presence of preoperative stroke. We believe that this difference stems from a combination of factors: arthroplasty usually requires more extensive surgical injury and longer anesthesia, intraoperative lateral recumbency adversely affects ventilatory function; and tissue damage caused by cement and prosthesis implantation is more significant (20, 58).

The SHAP analysis in this study has important value: through SHAP values, it is clarified that albumin, hemoglobin, age, and BNP are core predictive variables, and the interaction between variables is revealed (such as the synergistic risk of elevated BNP and decreased hemoglobin), providing clear targets for clinical intervention (such as prioritizing the improvement of nutritional status). Among the machine learning models, Logistic Regression and LDA have their own advantages: both have an AUC of 0.708, with accuracies of 0.722 and 0.718 respectively, and low Brier scores (0.199), indicating that the models not only have good discriminative ability but also more calibrated probability predictions, which are suitable for clinical promotion (Logistic Regression model has a simple structure and is easy to interpret; LDA model has strong stability in small samples). In contrast, other models have certain limitations: for example, the performance of Neural Network is poor (AUC = 0.507), which may be due to insufficient sample size that cannot support the training of complex models; although ensemble models such as Random Forest have high recall rates, their interpretability is poor, which limits their clinical application.

It should be noted that there is a certain difference between the training set AUC (0.9203) and the validation set AUC (0.7356) in this study, which reflects that the generalization ability of the model needs to be improved. This difference is mainly due to two aspects: first, this study is a single-center study with relatively single sample source, and the sample size of the validation set (about 210 cases) is small, which may lead to data heterogeneity; second, there may be bias in data distribution in retrospective studies, resulting in good fitting effect of the model on the training set, but reduced adaptability to new data. To address this issue, we plan to take the following improvement measures: Expand the sample size, plan to collect more case data through multi-center collaboration, and increase the external validation cohort; supplement the results of 5-fold cross-validation and hyperparameter tuning (basic tuning has been performed in this study, but not detailedly reported) to reduce overfitting; consider the use of regularization technology to further optimize the model. At the same time, we will objectively illustrate the applicable scope of the existing model to avoid overestimating its clinical applicability.

This study has several limitations. First, it was a single-center retrospective study with inherent selection and information biases, and a relatively limited sample size. Second, specific thresholds for laboratory indicators predicting perioperative pneumonia were not identified, nor were the dynamic changes of these indicators and their quantitative association with pneumonia occurrence tracked. Third, due to the retrospective nature, electronic medical records lacked systematic documentation of patients’ swallowing function assessments and detailed neurological function scores, excluding these stroke-related neurological indicators from the analysis; potential important variables such as microbiologic information were also not included. Lack of specific factors for stroke: Indicators such as stroke severity scores (such as NIHSS scores), previous history of aspiration pneumonia, and baseline cognitive function assessments (such as MMSE scores) were not included. These factors are closely related to the pathophysiological mechanisms of increased pneumonia risk in stroke patients (such as aspiration caused by dysphagia), which may affect the predictive performance of the model. Most importantly, this study did not systematically collect and analyze the dosage, duration and administration route of perioperative opioid analgesics, nor did it include standardized assessment indicators of swallowing mechanism in patients after stroke. In clinical practice, the use of opioid analgesics is closely related to the risk of postoperative respiratory failure and tracheal reintubation, and tracheal reintubation will further increase the incidence of pneumonia. Impaired swallowing function after stroke easily leads to aspiration during eating and drinking, which is an important independent inducement of perioperative pneumonia. The lack of these two types of information may cause a certain bias in the model’s prediction of pneumonia risk. Future studies need to incorporate them to optimize the model. Perioperative nursing process variables were not covered. These process variables may confuse the association between predictive variables and outcomes or become potential intervention targets. Their absence may limit the clinical applicability of the model. Subsequent studies should incorporate these variables for a more comprehensive analysis. Fourth, due to the retrospective design, the specific echocardiographic values of pulmonary arterial hypertension in all patients were not obtained, which may lead to diagnostic bias. Future prospective studies should incorporate objective measurement indicators to optimize the model. Finally, external validation was not performed, and the model’s efficacy in a broader population awaits further verification. Future prospective studies will design dedicated data collection forms to record swallowing function, neurological function recovery, and other indicators for model improvement.

## Conclusion

In conclusion, this study clarified the independent risk factors for perioperative pneumonia in elderly hip fracture combined with preoperative stroke patients through retrospective analysis, and established and validated a column-line graph prediction model. The model integrates multidimensional indicators such as age, BNP, hemoglobin, albumin, type of surgery and combined respiratory diseases, with high predictive efficacy, providing a practical tool for early clinical identification of high-risk patients. Through targeted interventions, such as optimizing respiratory function management, strengthening nutritional support and individualized surgical strategies, it is expected to reduce the incidence of pneumonia and improve the prognosis of patients. In the future, the model should be further validated and optimized through multicenter and large-sample prospective studies to promote its clinical translation and application.

## Data Availability

The raw data supporting the conclusions of this article will be made available by the author, without undue reservation.
